# Five‐Year Outcomes and Clinicopathological Correlations of Kidney Transplants From Deceased Donors With Diabetes

**DOI:** 10.1111/ctr.70698

**Published:** 2026-09-28

**Authors:** Marina Colella‐Santos, Pedro Henrique Pepato, Henrique Machado de Sousa Proença, Luiz Antonio Ribeiro de Moura, José Medina‐Pestana, Renato Demarchi Foresto, Lúcio Requião‐Moura

**Affiliations:** ^1^ Nephrology Division Federal University of São Paulo São Paulo Brazil; ^2^ Hospital Do Rim Fundação Oswaldo Ramos São Paulo Brazil

**Keywords:** Banff classification, deceased donor, diabetes mellitus, estimated glomerular filtration rate, graft survival, kidney transplantation, pre‐implantation biopsy

## Abstract

**Background:**

Kidneys from deceased donors with diabetes mellitus are refused because diabetes is considered a marker of lower organ quality. We evaluated 5‐year outcomes according to donor‐recipient diabetes status and, secondarily, the prognostic value of pre‐implantation histopathological findings among accepted diabetic donor kidneys.

**Methods:**

This retrospective single‐center cohort study included adult recipients of deceased‐donor kidney transplants performed between 2013 and 2017. Five‐year kidney function and death‐censored graft survival were compared according to donor diabetes status before and after 1:2 propensity score matching. A secondary exploratory analysis evaluated Banff chronicity parameters in pre‐implantation pathology reports.

**Results:**

Among 3058 recipients, 244 received kidneys from diabetic donors. These donors were older, more frequently hypertensive, and had higher KDPI than non‐diabetic donors. In unadjusted analyses, recipients of diabetic donor kidneys had lower eGFR and death‐censored graft survival. After matching, eGFR from years 1 to 5, eGFR slope, and death‐censored graft survival no longer differed between groups. Recipient diabetes did not significantly modify the association of donor diabetes with graft survival or longitudinal eGFR, although the D+/R+ subgroup was small (*n* = 39). Among 164 available pre‐implantation biopsy reports, diabetes‐related glomerular lesions were uncommon, chronic lesions were predominantly vascular and mild to moderate, and no Banff histological parameter independently predicted eGFR <30 mL/min/1.73 m^2^ or death‐censored graft loss at 5 years.

**Conclusions:**

After balancing baseline donor and recipient characteristics, donor diabetes was not associated with significantly lower 5‐year kidney function or death‐censored graft survival. In the secondary clinicopathological analysis, no individual Banff chronicity parameter independently predicted 5‐year outcomes.

**Abbreviations**
CI: confidence interval
CMV: cytomegalovirus
DM: diabetes mellitus
eGFR: estimated glomerular filtration rate
HCV: hepatitis C virus
HLA: human leukocyte antigen
KDPI: kidney donor profile index
KDRI: kidney donor risk index
mTOR: mammalian target of rapamycin
RR: relative risk

## 1 Introduction

Diabetes mellitus, a leading cause of chronic kidney disease worldwide, is increasingly common among deceased kidney donors. In organ allocation, donor diabetes is often considered a marker of reduced quality because it coexists with advanced age, hypertension, and cerebrovascular death [1, 2, 3]. Consequently, these kidneys are more frequently discarded, limiting donor utilization [2].

The impact of donor diabetes on transplant outcomes remains uncertain. Registry analyses report inferior graft survival in recipients of diabetic donor kidneys, whereas single‐center and matched studies suggest this risk largely reflects donor and recipient characteristics rather than diabetes itself [3, 4, 5]. Importantly, studies have reported poorer graft outcomes when diabetic donor kidneys are transplanted into diabetic recipients. Cohen et al. [3] identified a significant interaction between donor and recipient diabetes, with poorer graft outcomes among diabetic recipients of kidneys from diabetic donors. Singh et al. [6] similarly reported lower graft survival in the donor‐diabetic/recipient‐diabetic combination. More recently, Orsillo et al. [7], in a large Australia and New Zealand registry cohort, found that death‐censored graft survival was reduced specifically when both donor and recipient had diabetes. Nevertheless, transplantation with kidneys from diabetic donors may still provide a survival advantage compared with remaining on dialysis [8].

Our previous analysis of standard‐criteria deceased donors found lower unadjusted kidney function in recipients of diabetic donor kidneys, with no significant difference after propensity score matching [9]. The present cohort extends this assessment across the complete spectrum of deceased donors, including higher‐risk donor profiles. An additional unresolved question is whether pre‐implantation histology provides clinically useful prognostic information among diabetic donor kidneys that are accepted for transplantation. Overt diabetic nephropathy is uncommon in transplanted kidneys, whereas vascular and tubulointerstitial chronic lesions are frequently observed [10, 11]. If such lesions provide prognostic information, they could refine organ selection, recipient counseling, and risk‐adapted allocation.

We therefore evaluated 5‐year outcomes of kidney transplantation according to donor diabetes status in a large Brazilian deceased‐donor cohort, before and after propensity score matching, and examined whether recipient diabetes modified these associations. As a secondary exploratory analysis, we characterized Banff‐scored chronic lesions in diabetic donor kidneys with available pre‐implantation pathology reports and evaluated their associations with 5‐year kidney function and graft loss.

## 2 Methods

### 2.1 Study Design and Population

We conducted a retrospective single‐center cohort study including adult recipients of deceased‐donor kidney transplants performed at Hospital do Rim, São Paulo, Brazil, between January 2013 and December 2017, with follow‐up to 5 years. The institutional ethics committee approved the study (approval 5.320.890) and waived informed consent because of the retrospective design. Data were anonymized before analysis.

### 2.2 Eligibility Criteria

Adult recipients aged ≥18 years were eligible. Exclusion criteria were combined‐organ transplantation and loss to follow‐up within 1 month after transplantation. The primary analysis compared recipients of kidneys from donors with and without diabetes in the full cohort and after 1:2 propensity score matching. An additional exploratory analysis evaluated outcomes according to combined donor‐recipient diabetes status. A secondary exploratory clinicopathological analysis included recipients of kidneys from donors with diabetes for whom pre‐implantation pathology reports were available.

### 2.3 Data Sources and Variables

Clinical and transplant data were extracted from institutional electronic databases, including the center transplant system and the Collaborative Transplant Study dataset. Donor variables included age, sex, ethnicity, cause of death, donor classification, history of diabetes mellitus and hypertension, terminal serum creatinine, CMV serostatus, KDPI, and KDRI. KDPI was included as a donor risk summary because it has been evaluated in the Brazilian deceased‐donor kidney transplant setting [12].

Recipient variables included age, sex, ethnicity, primary CKD etiology, dialysis duration and modality, retransplantation, diabetes before transplantation, PRA, and CMV serostatus. Anti‐HLA antibodies were assessed using Luminex single‐antigen bead assays [13].

Transplant variables included cold ischemia time, HLA‐A, HLA‐B, and HLA‐DR compatibility, induction therapy, maintenance immunosuppression, delayed graft function, cytomegalovirus treatment during follow‐up, acute rejection, serum creatinine from years 1 to 5 after transplantation, graft loss, death, and loss to follow‐up.

### 2.4 Definitions and Clinical Protocols

eGFR was calculated using the 2021 CKD‐EPI creatinine equation [14]. Delayed graft function was defined as dialysis within the first week after transplantation, excluding dialysis during the first 24 h for isolated volume overload or hyperkalemia [15]. Acute rejection was biopsy‐proven according to Banff criteria or presumed when acute graft dysfunction was empirically treated for rejection with subsequent improvement [16]. Only brain‐death donors were used according to Brazilian transplantation regulations, and HCV‐positive donors were not used.

Pre‐implantation biopsy was part of routine institutional assessment of kidneys recovered from donors with diabetes. The rationale was to identify diabetes‐related histological changes and assess the severity of chronic parenchymal and vascular injury before organ acceptance. No prospectively defined or formally validated histological acceptance algorithm or numerical Banff cutoff was used. Instead, biopsy findings were interpreted together with the overall clinical donor profile by the transplant team. Kidneys showing moderate or severe diabetes‐related lesions or advanced chronic parenchymal injury were less likely to be accepted for transplantation.

Induction immunosuppression consisted of a single 3.0 mg/kg dose of anti‐thymocyte globulin administered within 24 h after transplantation. Maintenance immunosuppression included tacrolimus and prednisone plus a third agent selected according to immunological risk. Mycophenolic acid was preferentially used for recipients with higher immunological risk, whereas azathioprine or an mTOR inhibitor was used in selected recipients with lower immunological risk or according to donor and recipient characteristics [17].

### 2.5 Pre‐Implantation Biopsy Analysis

Pre‐implantation biopsies were performed in 238 of 244 kidneys from donors with diabetes (97.5%). Pathology reports were available for 164 biopsies (68.9% of biopsied kidneys) and constituted the clinicopathological cohort. Samples were fixed in 10% buffered formalin, rapidly processed, sectioned at 3 µm, and stained with hematoxylin and eosin, Masson's trichrome, Jones’ silver, and periodic acid‐Schiff. Frozen sections were not used. Available reports were reviewed for Banff chronicity lesions, including glomerular basement membrane changes, mesangial matrix expansion, interstitial fibrosis, tubular atrophy, vascular fibrous intimal thickening, and arteriolar hyalinosis [18].

### 2.6 Outcomes

The primary outcome was kidney graft function over time, assessed by eGFR from year 1 through year 5 after transplantation. The secondary outcome was death‐censored graft survival. In the clinicopathological cohort, we evaluated the association of Banff‐scored lesions with two 5‐year outcomes: eGFR <30 mL/min/1.73 m^2^ and death‐censored graft loss.

### 2.7 Statistical Analysis

Analyses were performed at the recipient level. Continuous variables were assessed for normality, summarized as medians and interquartile ranges, and compared with the Mann‐Whitney U test. Categorical variables were summarized as counts and percentages and comparing using chi‐square or Fisher exact tests, as appropriate. Longitudinal eGFR from years 1 to 5 was analyzed using linear generalized estimating equations including time, donor diabetes status, and the time‐by‐group interaction. Pairwise comparisons were adjusted using the Bonferroni method. Missing eGFR values were imputed using last observation carried forward after death or loss to follow‐up as zero after graft failure.

Death‐censored graft survival was estimated using Kaplan‐Meier method and compared with the log‐rank test. To reduce confounding in the DM+ versus DM‐ comparison, 1:2 nearest‐neighbor propensity score matching was performed using donor age and hypertension, cause of brain death, and HLA‐DR compatibility. Matching was conducted with MatchIt in R [19]. Covariate balance selected and propensity‐score distributions were reassessed after matching. Pairwise log‐rank comparisons among the four donor‐recipient diabetes groups were adjusted for multiple testing using the Holm method.

Effect modification by recipient diabetes was evaluated in the full cohort by classifying recipients as D‐/R‐, D‐/R+, D+/R‐, or D+/R+. Cox proportional hazards regression included donor diabetes, recipient diabetes, their interaction, donor age, donor hypertension, cause of brain death, recipient age, retransplantation, and HLA‐DR compatibility. Longitudinal eGFR was assessed with generalized estimating equations including donor diabetes, recipient diabetes, time, and the corresponding interactions, adjusted for the same covariates. This analysis was performed in the full cohort rather than after additional matching to preserve the small D+/R+ subgroup and directly test effect modification.

In the biopsy subgroup, logistic regression with backward stepwise selection evaluated eGFR <30 mL/min/1.73 m^2^ at 5 years. Variables with *p* < 0.20 in univariable analyses entered the initial model. Death‐censored graft loss was evaluated with Cox proportional hazards regression. Statistical analyses were performed using SPSS version 29.0 (IBM Corp., Armonk, NY, USA) and R version 4.1.3, with two‐sided *p* < 0.05 considered statistically significant.

## 3 Results

Of 3059 screened kidney transplants, one recipient was excluded because follow‐up was shorter than one month. The final cohort included 3058 recipients, categorized according to donor diabetes status as DM+ (*n* = 244) and DM‐ (*n* = 2,814). The prevalence of diabetes among donors was 8.0%.

### 3.1 Baseline Characteristics

DM+ donors were older (56 vs. 48 years, *p* < 0.001), more frequently hypertensive (79.9% vs. 37.3%, *p* < 0.001), more often died from cerebrovascular disease (75.4% vs. 59.2%, *p* < 0.001), had higher KDPI (92.5% vs. 67.0%, *p* < 0.001), and were more frequently expanded‐criteria donors (70.1% vs. 34.0%, *p* < 0.001). No differences were observed in other donor characteristics, including terminal serum creatinine.

Recipients of DM+ kidneys were slightly older (51.1 vs. 49.5 years, *p* = 0.02) and less often retransplanted (3.3% vs. 7.7%, *p* = 0.01). Although HLA matching differed modestly, cold ischemia time and induction therapy were similar. Maintenance immunosuppression with tacrolimus, prednisone, and mycophenolate sodium was more common among recipients of DM+ kidneys (68.4% vs. 55.4%, *p* < 0.001) (Table 1).

**TABLE 1:** Baseline donor, recipient, transplant, immunosuppression, and post‐transplant characteristics according to donor diabetes status.

Variable	General	DM‐	DM+	*p*
	*N*=3058	*N*=2814	*N*=244	
**Donors’ characteristics**				
Age, years	49.0 (37.0–57.0)	48.0 (36.0–56.0)	56.0 (49.0–62.0)	<0.001
Male, *n* (%)	1737 (56.8)	1611 (57.2)	126 (51.6)	0.05
White, *n* (%)	1632 (53.4)	1494 (53.1)	138 (56.6)	0.16
Brain death cause, *n* (%)				<0.001
Stroke	1851 (60.5)	1667 (59.2)	184 (75.4)	
Trauma	936 (30.6)	895 (31.2)	41 (16.8)	
Anoxia	151 (4.9)	165 (5.9)	16.6 (13.7)	
Others	90 (2.9)	87 (3.1)	3 (1.2)	
Arterial hypertension, *n* (%)	1246 (40.7)	1051 (37.3)	195 (79.9)	<0.001
Last creatinine, mg/dL	1.50 (1.0–2.6)	1.50 (0.91–2.6)	1.52 (1.52–2.40)	0.78
KDPI, %	70 (44–87)	67 (42–85)	92,5 (82–98)	<0.001
KDRI	1.20 (0.95–1.51)	1.18 (0.92–1.47)	1.61 (1.40–2.03)	<0.001
ECD, *n* (%)	1128 (36.9)	957 (34.0)	171 (70.1)	<0.001
**Recipients’ characteristics**				
Age, years	49.6 (39.2–58.6)	49.5 (38.9–58.4)	51.1 (42.3–60.5)	0.02
Male, *n* (%)	1872 (61.2)	1712 (60.8)	160 (65.6)	0.08
White, (%)	1391 (45.5)	1273 (45.2)	118 (48.4)	0.19
CKD etiology, *n* (%)				0.60
Arterial hypertension	1516 (49.6)	1395 (49.6)	121 (49.6)	
Glomerulonephritis	553 (17.4)	491 (17.4)	42 (17.2)	
Diabetes mellitus	482 (15.2)	444 (15.8)	38 (15.3)	
ADPKD	236 (7.7)	212 (7.5)	24 (9.8)	
Urological	195 (6.4)	182 (6.5)	13 (5.3)	
Lithiasis	41 (1.3)	37 (1.3)	4 (1.6)	
Undetermined	55 (1.8)	53 (1.9)	2 (0.8)	
Diabetes Mellitus, *n* (%)	621 (20.3)	582 (20.7)	39 (16.0)	0.32
RRT time, months	36.9 (22.3–70.2)	39.8 (22.3–70.2)	37.0 (21.0–70.0)	0.50
Hemodialysis as RRT, *n* (%)	2192 (96.1)	2704 (96.4)	235 (96.6)	0.32
Retransplantation, *n* (%)	226 (7.4)	218 (7.7)	8 (3.3)	0.01
Qualitative PRA, *n* (%)				
Class I > 0%	773 (25.3)	715 (25.4)	58 (23.8)	0.32
Class II > 0%	348 (11.4)	324 (11.5)	24 (9.8)	0.25
Positive CMV IgG, *n* (%)	2878 (94.1)	2651 (94.2)	227 (93.0)	0.27
**Transplantation and immunosuppression**				
Zero HLA‐A mismatch, *n* (%)	572 (18.7)	541 (19.2)	31 (12.7)	<0.001
Zero HLA‐B mismatch, *n* (%)	651 (21.3)	609 (21.6)	42 (17.2)	0.32
Zero HLA‐DR mismatch, *n* (%)	2617 (85.6)	2399 (85.3)	218 (89.3)	0.04
CIT, hours	23 (19–28)	23 (19–28)	23 (19–27)	0.88
Thymoglobulin induction, *n* (%)	3058 (98.6)	2773 (98.5)	242 (99.2)	0.63
Immunosuppressive regimen, *n* (%)				<0.001
TAC/MPA	1726 (56.4)	1560 (55.4)	166 (68.0)	
TAC/AZA	991 (32.4)	943 (33.5)	48 (19.7)	
TAC/ mTORi	335 (11.0)	306 (10.9)	29 (11.9)	
Other	7 (0.2)	6 (0.2)	1 (0.4)	
**Events after transplantation**				
DGF incidence, *n* (%)	1862 (60.9)	1720 (61.1)	142 (58.2)	0.37
CMV infection incidence, *n* (%)	1387 (45.4)	1265 (45.0)	122 (50.0)	0.13
AR, *n* (%)	648 (21.2)	592 (21.0)	56 (23.0)	0.26
Death, *n* (%)	371 (12.1)	354 (12.6)	17 (7.0)	0.01
Follow‐up loss, *n* (%)	239 (7.8)	223 (7.9)	16 (6.6)	0.06

Abbreviations: ADPKD, Autosomal dominant polycystic kidney disease; AR, acute rejection; AZA, azathioprine; CIT, cold ischemia time; CKD, chronic kidney disease; CMV, cytomegalovirus; DGF, delayed graft function; KDPI, kidney donor profile index; MPA, mycophenolate acid; mTORi, mammalian target of rapamycin inhibitors; PRA, panel reaction against antibodies; RRT, renal replacement therapy; TAC, tacrolimus.

### 3.2 Kidney Function and Graft Survival in the Overall Cohort

A total of 15,290 eGFR measurements collected from year 1 to 5 were analyzed. Mean eGFR declined from 42.0 mL/min/1.73 m^2^ (95% CI, 40.8 to 43.3) at year 1 to 35.0 mL/min/1.73 m^2^ (95% CI, 33.5 to 36.5) at year 5, corresponding to a mean decline of 7.0 mL/min/1.73 m^2^ (95% CI, 5.68 to 8.32, *p* < 0.001 for time effect, Bonferroni adjusted).

When stratified by donor diabetes status, 14,070 repeated measurements were available for recipients of DM‐ kidneys and 1220 for recipients of DM+ kidneys. Across follow‐up, recipients of DM+ kidneys had lower eGFR, with an overall adjusted mean difference of −9.26 mL/min/1.73 m^2^ (95% CI, −11.90 to −6.63, *p* < 0.001, Bonferroni adjusted). At year 1, mean eGFR was 37.8 mL/min/1.73 m^2^ (95% CI, 35.4 to 40.1) in recipients of DM+ kidneys and 46.3 mL/min/1.73 m^2^ (95% CI, 45.5 to 47.1) in recipients of DM‐ kidneys (mean difference −8.48, 95% CI, −12.62 to −4.35, *p *< 0.001, Bonferroni adjusted). At year 5, mean eGFR was 30.3 mL/min/1.73 m^2^ (95% CI, 27.5 to 33.0) in recipients of DM+ kidneys and 39.8 mL/min/1.73 m^2^ (95% CI, 38.8 to 40.7) in recipients of DM‐ kidneys (mean difference −9.52, 95% CI, −14.40 to −4.61, *p* < 0.001, Bonferroni adjusted). Despite this persistent difference in absolute function, the decline from year 1 to year 5 was similar between groups (−7.52 vs. −6.48 mL/min/1.73 m^2^; *p* = 0.52; Figure 1).

**FIGURE 1:**
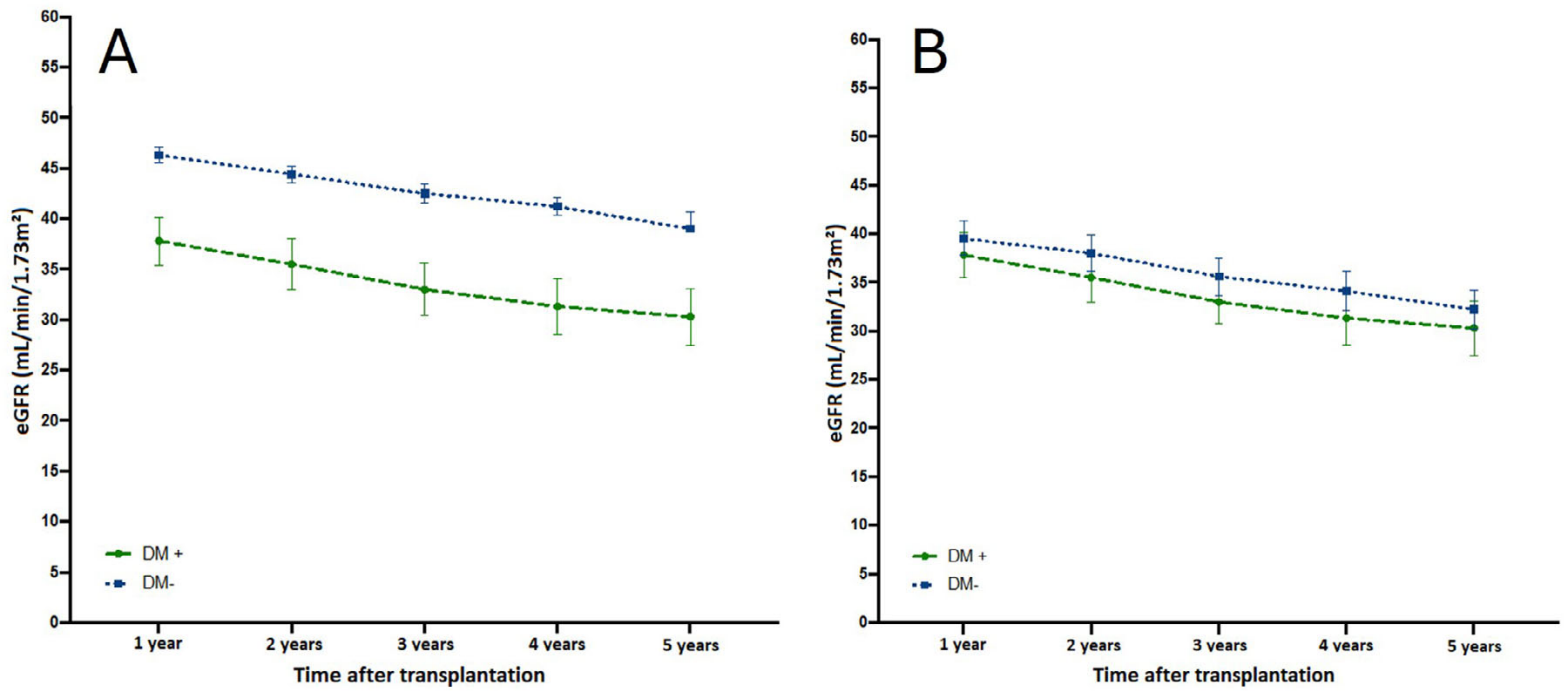
Mean estimated glomerular filtration rate over 5 years according to donor diabetes status in the overall cohort (A) and after propensity score matching (B). Error bars represent 95% confidence intervals. DM+, donor with diabetes mellitus; DM‐, donor without diabetes mellitus.

Death‐censored graft survival also differed in the unadjusted analysis (Figure 2). At year 1, death‐censored graft survival was 93.7% for recipients of DM+ donor kidneys and 95.1% for recipients of DM‐ donor kidneys. At year 5, death‐censored graft survival rates were 76.1% and 85.0%, respectively (log rank *p* < 0.001; Figure 2).

**FIGURE 2:**
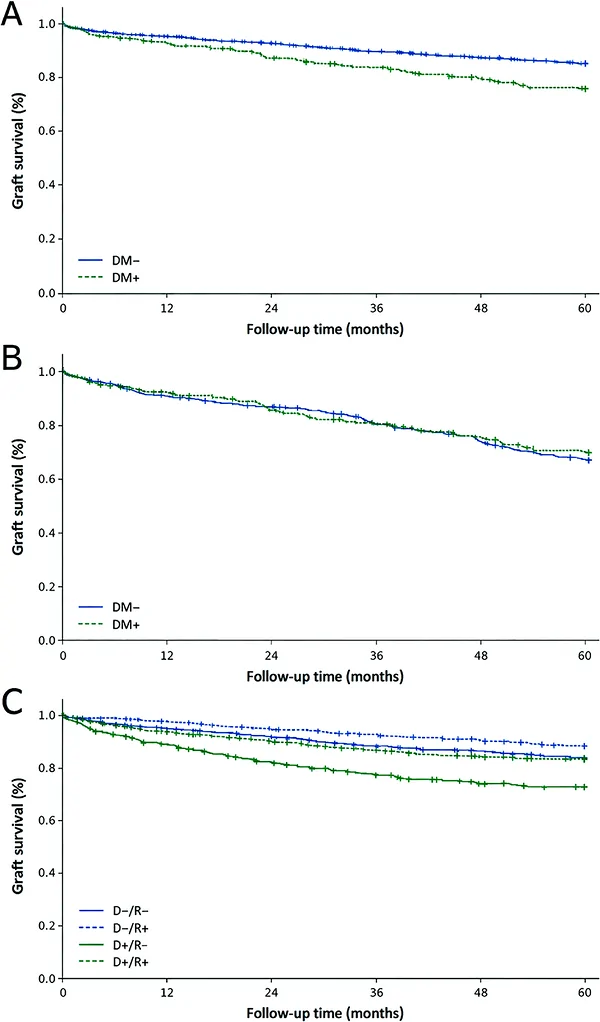
Death‐censored graft survival according to donor diabetes status in the overall cohort (A), after propensity score matching (B), and according to combined donor and recipient diabetes status in the overall cohort (C). D‐/R‐, nondiabetic donor/nondiabetic recipient; D‐/R+, nondiabetic donor/diabetic recipient; D+/R‐, diabetic donor/nondiabetic recipient; D+/R+, diabetic donor/diabetic recipient.

### 3.3 Outcomes after Propensity Score Matching

Propensity score matching included 244 DM+ and 488 DM‐ recipients. Matching accounted for donor age and hypertension, cause of brain death, and HLA‐DR compatibility. Propensity score distribution is shown in Figure 3, and donor, recipient, and transplant characteristics after matching are presented in Table 2. The prespecified matching covariates and most baseline characteristics were balanced, although KDPI and KDRI remained higher among DM+ donors.

**FIGURE 3:**
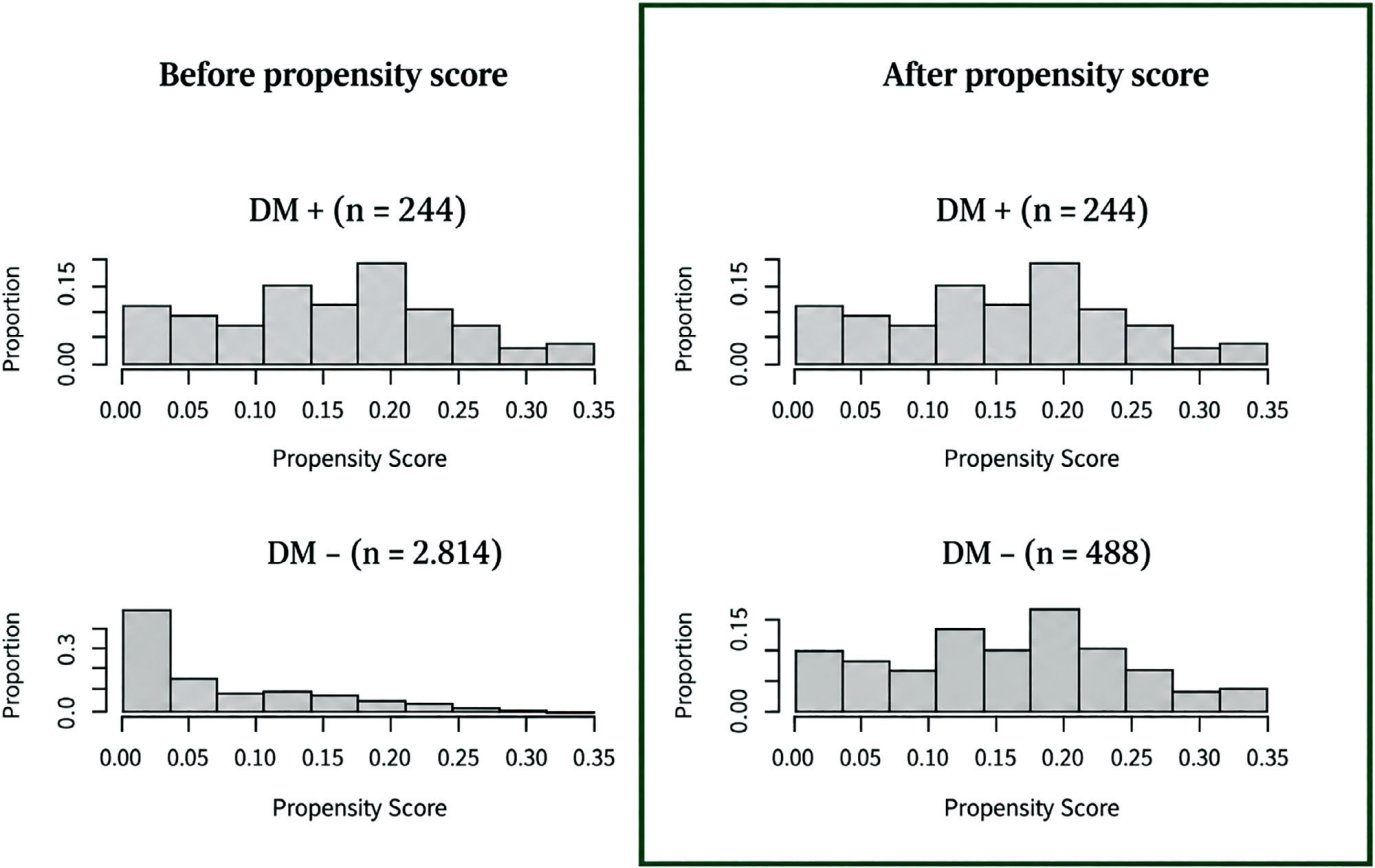
Propensity score distribution according to donor diabetes status before and after 1:2 propensity score matching. DM+, donor with diabetes mellitus; DM‐, donor without diabetes mellitus.

**TABLE 2:** Donor, recipient, and transplant characteristics after propensity score matching according to donor diabetes status.

Variable	DM‐	DM+	*p*
	*N*=488	*N*=244	
**Donors’ characteristics**			
Age, years	56.0 (49.0–63.0)	56 (49.0–62.0)	0.24
Male, *n* (%)	253 (51.8)	126 (51.6)	0.51
White, *n* (%)	272 (55.7)	138 (56.6)	0.30
Arterial hypertension, *n* (%)	385 (78.9)	195 (79.9)	0.42
Brain death cause, *n* (%)			0.26
Stroke	366 (75.0)	184 (75.4)	0.26
Trauma	93 (19.1)	41 (16.8)	
Anoxia	22 (4.5)	16 (6.6)	
Others	5 (1.4)	3 (1.2)	
Last creatinine, mg/dL	1.50 (1.0–2.6)	1.52 (1.0–2.40)	
KDPI, %	87.0 (71.0–94.7)	92.5 (82.0–98.0)	<0.01
KDRI	1.50 (1.23–1.77)	1.64 (1.40–2.03)	<0.01
ECD, *n* (%)	323 (66.2)	171 (70.1)	0.17
**Recipients’ characteristics**			
Age, years	51.4 (41.3–60.2)	51.1 (42.3–60.5)	0.15
Male, *n* (%)	314 (60.4)	160 (65.6)	0.40
White, (%)	230 (47.1)	118 (48.4)	0.57
CKD etiology, *n* (%)			0.35
Arterial hypertension	249 (51.0)	121 (49.6)	
Glomerulonephritis	70 (14.3)	42 (17.2)	
Diabetes mellitus	73 (15.0)	38 (15.3)	
ADPKD	38 (7.8)	24 (9.8)	
Urological	41 (8.4)	13 (5.3)	
Lithiasis	5 (1.0)	4 (1.6)	
Undetermined	12 (2.5)	2 (0.8)	
RRT time, months	38.5 (21.9–68.4)	37.0 (21.0–70.0)	0.39
Hemodialysis as RRT, *n* (%)	466 (95.5)	235 (96.6)	0.63
Retransplantation, *n* (%)	12 (2.5)	8 (3.3)	0.34
Qualitative PRA, *n* (%)			
Class I > 0%	96 (19.7)	58 (23.8)	0.12
Class II > 0%	42 (8.6)	24 (9.8)	0.34
Positive CMV IgG, *n* (%)	458 (93.9)	227 (93.0)	0.39
**Transplants characteristics**			
Zero HLA‐A mismatch, *n* (%)	50 (10.2)	31 (12.7)	0.72
Zero HLA‐B mismatch, *n* (%)	94 (19.3)	42 (17.2)	0.55
Zero HLA‐DR mismatch, *n* (%)	429 (87.9)	218 (89.3)	0.58
CIT, hours	23 (19–28)	23 (19–27)	0.67
Thymoglobulin induction, *n* (%)	446 (99.6)	242 (99.2)	0.41
Immunosuppressive regimen, *n* (%)			0.51
TAC/MPA	352 (72.1)	166 (68.0)	
TAC/AZA	91 (18.6)	48 (19.7)	
TAC/ mTORi	45 (9.2)	29 (11.9)	
Other	0 (0.0)	1 (0.4)	

Abbreviations: ADPKD, Autosomal dominant polycystic kidney disease; AZA, azathioprine; CIT, cold ischemia time; CKD, chronic kidney disease; CMV, cytomegalovirus; KDPI, kidney donor profile index; MPA, mycophenolate acid; mTORi, mammalian target of rapamycin inhibitors; N, number; PRA, panel reaction against antibodies; RRT, renal replacement therapy; TAC, tacrolimus.

Among 3660 eGFR measurements in the matched cohort, eGFR declined from year 1 to year 5, with a mean reduction of 7.36 mL/min/1.73 m^2^ (95% CI, 5.94 to 8.79, *p* < 0.001). In contrast, no significant difference in mean eGFR was detected between groups, with an overall adjusted mean difference of −2.30 mL/min/1.73 m^2^ (95% CI, −5.37 to 0.78, *p* = 0.14). At year 1, mean eGFR was 37.8 mL/min/1.73 m^2^ (95% CI, 35.4 to 40.1) in DM+ and 39.5 mL/min/1.73 m^2^ (95% CI, 37.8 to 41.3) in DM‐ group (mean difference −1.75, 95% CI, −6.62 to 3.11, *p* = 0.99, Bonferroni adjusted). At year 5, mean eGFR was 30.3 mL/min/1.73 m^2^ (95% CI, 27.5 to 33.1) in DM+ and 32.2 mL/min/1.73 m^2^ (95% CI, 30.1 to 34.2) in DM‐ group (mean difference −1.91, 95% CI, −7.67 to 3.85, *p* = 0.99, Bonferroni adjusted). There was no significant time‐by‐group interaction (*p* = 0.57; Figure 1).

After matching, death‐censored graft survival at years 1 and 5 was 93.7% and 76.1% in DM+, and 93.1% and 78.9% in DM‐ group (log‐rank *p* = 0.35; Figure 2). No significant differences were observed between groups in delayed graft function (58.2% vs. 63.5%, *p* = 0.22), CMV infection (50.0% vs. 53.9%, *p* = 0.32), acute rejection (23.0% vs. 21.0%, *p* = 0.44), loss to follow‐up (6.6% vs. 7.0%, *p* = 0.48), or mortality (7.0% vs. 12.3%, *p* = 0.16).

### 3.4 Outcomes According to Donor and Recipient Diabetes Status

The D‐/R‐, D‐/R+, D+/R‐, and D+/R+ groups included 2232, 582, 205, and 39 recipients, respectively. Five‐year death‐censored graft survival was 84.3%, 86.6%, 73.9%, and 84.1%, respectively (global log‐rank *p* < 0.001; Figure 2). After Holm adjustment for pairwise comparisons, survival was lower in D+/R‐ than in D‐/R‐ (*p* = 0.002) and D‐/R+ (*p* < 0.001). D+/R+ did not differ significantly from any other group, including D+/R‐ (adjusted *p* = 0.80).

In adjusted Cox regression, the donor diabetes‐by‐recipient diabetes interaction was not statistically significant (*p* = 0.205). Compared with D‐/R‐, adjusted HRs for death‐censored graft loss were 1.02 (95% CI, 0.78–1.33) for D‐/R+, 1.43 (95% CI, 1.05–1.95) for D+/R‐, and 0.82 (95% CI, 0.36–1.84) for D+/R+. Only six death‐censored graft losses occurred among the 39 D+/R+ recipients. Mean eGFR declined from 44.7 to 38.2 mL/min/1.73 m^2^ in D‐/R‐, from 43.6 to 37.2 in D‐/R+, from 36.5 to 28.5 in D+/R‐, and from 38.2 to 33.6 in D+/R+ between years 1 and 5. Neither the donor diabetes‐by‐recipient diabetes interaction (*p* = 0.311) nor the three‐way time‐by‐donor diabetes‐by‐recipient diabetes interaction (*p* = 0.100) was statistically significant.

### 3.5 Pre‐Implantation Biopsy Findings Among Diabetic Donor Kidneys

Among the 244 transplanted kidneys from donors with diabetes, 238 (97.5%) underwent pre‐implantation biopsy. Pathology reports were available for 164 of these biopsied kidneys (68.9%; 67.2% of all transplanted diabetic donor kidneys), which comprised the clinicopathological analysis cohort. Donor, recipient, and transplant characteristics for this subgroup are shown in Table S1.

Glomerular basement membrane changes were absent (cg = 0 in 100%). Mesangial matrix expansion was rare (mm = 1–2 in 1.8%). Tubular atrophy and interstitial fibrosis were predominantly absent or mild (ct = 0 in 70.7% and ci = 0 in 69.5%), whereas fibrous intimal thickening and arteriolar hyalinosis were common, occurring in 63.4% and 59.1% of cases, respectively (Table S2).

Delayed graft function occurred in 58.5% of recipients, CMV infection or disease requiring antiviral therapy in 49.9%, and biopsy‐proven acute rejection in 17.1%. Five‐year cumulative mortality was 16.5%.

### 3.6 Outcomes in the Biopsy Subgroup

At year 5, 83 recipients (50.6%) had eGFR <30 mL/min/1.73 m^2^. Univariable donor, recipient, transplant, immunosuppression, and histological comparisons are provided in Tables S3–S6. Variables with *p* < 0.20 were considered for multivariable modeling. No Banff chronicity parameter independently predicted eGFR <30 mL/min/1.73 m^2^.

In the final logistic model, tacrolimus plus an mTOR inhibitor, compared with tacrolimus plus mycophenolate, was associated with higher odds of eGFR <30 mL/min/1.73 m^2^ (OR, 2.09; 95% CI, 1.15–3.78; *p* = 0.015; Table 3).

**TABLE 3:** Multivariable analysis of factors associated with eGFR <30 mL/min/1.73 m^2^ at 5 years among recipients of kidneys from donors with diabetes mellitus.

	OR	95% CI	*p*
Donor age (per year)	1.02	0.99–1.05	0.10
Immunosuppression			
Pred + TAC + MPS (ref.)	1.00	–	–
Pred + TAC + AZA	1.20	0.64–2.24	0.57
Pred + TAC + mTORi	2.09	1.15–3.78	0.01
Other	4.50	0.60–33.8	0.14

*Note:* Variables removed by backward stepwise selection: recipient age (step 2), dialysis time (step 3), PRA class I (step 4), tubular atrophy (ct) (step 5), and dialysis modality (step 6).

Abbreviations: AZA, azathioprine; CI, confidence interval; MPS, mycophenolate sodium; mTORi, mammalian target of rapamycin inhibitors; OR, oddis ratio; Pred, prednisone; TAC, tacrolimus.

Twenty‐seven recipients (16.5%) experienced death‐censored graft loss by year 5. Univariable donor, recipient, transplant‐related, immunosuppression, and histological comparisons are shown in Tables S7–S10. Donor age, donor hypertension, and vascular fibrous intimal thickening were entered into the Cox model according to the prespecified selection strategy. No histological parameter was independently associated with death‐censored graft loss (Table S11).

## 4 Discussion

In this large Brazilian deceased‐donor cohort, donor diabetes was associated with lower kidney function and death‐censored graft survival in unadjusted analyses, but these differences were substantially attenuated after propensity score matching. The rate of eGFR decline from years 1 through 5 was also similar according to donor diabetes status. Recipient diabetes did not significantly modify graft survival or longitudinal eGFR, although the D+/R+ subgroup was small. In the secondary clinicopathological analysis, no individual Banff chronicity parameter independently predicted 5‐year outcomes. Together, these findings support use of carefully selected kidneys from donors with diabetes while emphasizing assessment of the complete donor‐recipient risk profile rather than donor diabetes as an isolated exclusion criterion. The clinicopathological component should be interpreted as an exploratory analysis of selected transplanted kidneys with available pathology data, not as a validation of biopsy as a stand‐alone acceptance tool.

Diabetic donors in our cohort had a marked clustering of adverse characteristics, including older age, hypertension, cerebrovascular death, and expanded‐criteria status. Similar clustering has been described in previous studies and can confound the apparent effect of diabetes in crude comparisons [20]. This is relevant because donor age, hypertension, and vascular disease may independently influence nephron mass, chronic histological injury, and subsequent graft function. In the full cohort, recipients of DM+ kidneys had lower eGFR from the first year onward and lower graft survival at 5 years. After balancing major baseline differences, however, absolute eGFR and death‐censored graft survival were no longer significantly different, even though KDPI and KDRI remained higher among DM+ donors. Propensity‐matched and adjusted studies have similarly reported attenuation of risk after accounting for donor and recipient characteristics [21, 22].

The functional trajectory provides additional information beyond a single eGFR value. In the overall cohort, recipients of DM+ kidneys began with lower eGFR and maintained this difference through year 5, but the rate of decline was not steeper. After matching, both the level of function and the longitudinal slope were comparable. Previous studies by Becker et al. and Singh et al. [4, 6] also reported lower renal function in recipients of diabetic donor kidneys, although analyses were based primarily on serum creatinine or discrete function estimates rather than repeated eGFR trajectories. Our findings therefore suggest that selected diabetic donor kidneys may enter the post‐transplant period with lower functional reserve without necessarily undergoing accelerated medium‐term decline. This distinction is clinically relevant when discussing expected graft performance and may help avoid interpreting a lower initial eGFR as evidence of progressive donor‐derived diabetic injury.

A similar pattern was observed for graft survival. The unadjusted disadvantage associated with diabetic donor kidneys disappeared after matching. This supports the possibility that much of the observed excess risk in routine cohorts reflects the broader clinical phenotype of diabetic donors. Nevertheless, large registry analyses with longer follow‐up have reported inferior long‐term graft survival among recipients of kidneys from diabetic donors [3, 5]. Late adverse effects may therefore emerge in selected populations or with longer observation. Importantly, the clinical decision is not simply between a diabetic and a non‐diabetic donor kidney. For many candidates, the relevant alternative is remaining on dialysis while waiting for another offer. Previous work has demonstrated a survival advantage of accepting a diabetic donor kidney compared with remaining on the waiting list, reinforcing the need to consider this counterfactual in organ acceptance decisions [8].

Recipient diabetes may further modify the risk associated with donor diabetes. Cohen et al. [3] identified a significant donor‐recipient diabetes interaction, Singh et al. [6] reported the lowest graft survival when both donor and recipient had diabetes, and Orsillo et al. [7] observed reduced death‐censored graft survival specifically in D+/R+ recipients in the ANZDATA Registry. We did not reproduce that pattern. The lowest observed 5‐year graft survival occurred in D+/R‐ recipients, and neither Cox regression nor longitudinal GEE demonstrated a significant donor‐by‐recipient diabetes interaction. However, only 39 recipients belonged to the D+/R+ group and only six experienced death‐censored graft loss. The wide confidence intervals and limited number of events preclude excluding clinically meaningful effect modification. Our findings should therefore be interpreted as failure to demonstrate an interaction in this cohort rather than evidence that recipient diabetes is irrelevant.

Beyond clinical graft outcomes, the recipient metabolic environment may influence the evolution of pre‐existing diabetic lesions in the transplanted kidney. Evidence remains limited and heterogeneous. Harada et al. [23] reported improvement of early diabetic nephropathy lesions in three diabetic donor kidneys transplanted into non‐diabetic recipients with good glycemic control. In contrast, Lee et al. [24], using paired protocol biopsies obtained early and at 1 year after transplantation, found that diabetic nephropathy class remained stable in most recipients and that histological progression was not significantly associated with recipient diabetes or fasting glucose control. Other small series have reported both regression and progression of donor‐derived diabetic lesions [25]. Because our cohort lacked systematic post‐transplant protocol biopsies and longitudinal measures of glycemic control, the donor‐recipient diabetes analysis addresses clinical outcomes but cannot determine whether recipient diabetes or post‐transplant glycemic exposure modified the histological course of pre‐existing diabetic injury.

Pre‐implantation histology in our transplanted diabetic donor kidneys was characterized mainly by vascular and mild tubulointerstitial chronicity rather than overt glomerular diabetic lesions, consistent with previous clinicopathological report .[10, 11]. This distribution is expected in a program where biopsy contributes to organ assessment because kidneys with advanced diabetic or chronic parenchymal injury are less likely to be transplanted. Within this selected population, no Banff lesion independently predicted eGFR <30 mL/min/1.73 m^2^ or death‐censored graft loss at 5 years. This differs from reports associating arteriolar hyalinosis, interstitial fibrosis and tubular atrophy, glomerular hypertrophy, or other chronic lesions with inferior graft outcomes [11, 26]. Differences in lesion prevalence, biopsy availability, pathological classification, organ‐selection practices, follow‐up duration, and event rates may explain part of this divergence.

Duration and severity of donor diabetes may also contribute to heterogeneity. Gilbert et al. found that donor diabetes lasting ≥6 years was associated with shorter death‐censored graft survival, supporting the concept that donor diabetes should not be treated as a simple binary exposure [11]. Longer metabolic exposure may reflect a greater cumulative burden of vascular, tubulointerstitial, and diabetes‐related kidney injury. Reliable information on duration of donor diabetes, HbA1c, proteinuria, and albuminuria was not systematically available in our cohort. We therefore could not determine whether the acceptable outcomes observed here partly reflect preferential transplantation of kidneys from donors with shorter or less severe diabetes exposure.

The association between mTOR inhibitor‐based immunosuppression and eGFR <30 mL/min/1.73 m^2^ at 5 years should be interpreted cautiously. Only a small proportion of the biopsy cohort received this regimen, and treatment selection was not randomized. mTOR inhibitors may have been preferentially chosen for recipients with particular donor profiles, clinical risks, or post‐transplant complications. Residual confounding and confounding by indication are therefore plausible. This result should be considered hypothesis‐generating rather than evidence of a causal adverse effect of mTOR inhibition in recipients of diabetic donor kidneys.

Several limitations should be considered. First, the retrospective single‐center design limits causal inference and may restrict generalizability. Second, the clinicopathological analysis was restricted to transplanted diabetic donor kidneys, and pathology reports were available for 164 of 238 biopsied kidneys, introducing potential selection and information bias. Because kidneys with advanced histological injury were less likely to be accepted, the biopsy cohort does not represent the complete histological spectrum of kidneys recovered from diabetic donors. Third, chronicity was assessed by routine pre‐implantation light microscopy and could not be uniformly classified using the Renal Pathology Society system for diabetic nephropathy [27]. Fourth, reliable donor diabetes duration, metabolic‐control measures, proteinuria, and albuminuria were unavailable. Fifth, the limited number of events in the biopsy and D+/R+ subgroups reduced statistical power. Finally, absent post‐transplant protocol biopsies and longitudinal glycemic data prevented direct assessment of progression or regression of donor‐derived diabetic lesions.

The study also has several strengths. It includes a large consecutive cohort from a high‐volume transplant center in a middle‐income country, evaluates repeated kidney‐function measurements rather than isolated creatinine values, and uses propensity score matching to address major baseline differences. The additional donor‐recipient analysis directly evaluates an effect modifier highlighted in contemporary registry studies. The clinicopathological component integrates donor histology with clinical outcomes and clearly defines the selected population to which the findings apply. Taken together, these analyses suggest that carefully selected kidneys from donors with diabetes can provide acceptable medium‐term outcomes and that organ acceptance should incorporate the overall clinical and histological context rather than donor diabetes alone.

## 5 Conclusion

In this large single‐center cohort, differences in 5‐year kidney function and death‐censored graft survival associated with donor diabetes were attenuated after balancing baseline donor and recipient characteristics, and longitudinal eGFR decline was similar between groups. No significant effect modification by recipient diabetes was detected, although the small D+/R+ subgroup limits this inference. Among selected diabetic donor kidneys with available pre‐implantation pathology reports, no Banff chronicity parameter independently predicted 5‐year outcomes. These findings support careful use of kidneys from donors with diabetes based on integrated donor‐recipient risk assessment and histological context rather than exclusion according to donor diabetes status alone.

## Author Contributions

**Marina Colella‐Santos**: study concept and design, data collection, data interpretation, statistics, drafting article. **Pedro Henrique Pepato**: data collection, statistics, critical revision of article. **Henrique Machado de Sousa Proença**: data collection, data interpretation, critical revision of article. **Luiz Antonio Ribeiro de Moura**: data collection, data interpretation, critical revision of article. **José Medina Pestana**: study concept and design, data interpretation, critical revision of article. **Renato Demarchi Foresto**: study concept and design, data interpretation, drafting article, critical revision of article. **Lúcio Requião‐Moura**: study concept and design, data interpretation, critical revision of article.

## Funding

This study received financial support from the Fundação Oswaldo Ramos.

## Disclosures

ChatGPT (OpenAI) and Grammarly were used to support the writing and editing of this manuscript by helping improve clarity, grammar, and phrasing.

## Conflicts of Interest

The authors declare no conflicts of interest.

## Supporting information




**Supporting Information**: ctr70698‐sup‐0001‐SuppMat.docx

## Data Availability

The data that support the findings of this study are available from the corresponding author upon reasonable request.
