# Supplementary material for: Five‐Year Outcomes and Clinicopathological Correlations of Kidney Transplants From Deceased Donors With Diabetes
Source: Clin Transplant. 2026 Sep 28;40(10):e70698. doi: 10.1111/ctr.70698 (PMC13619057; doi:10.1111/ctr.70698)
Supplement: Supplementary file 1 [file CTR-40-e70698-s001.docx]

**Supplementary Table 1.** Donor, recipient, and transplant characteristics among recipients with available pre-implantation kidney biopsy reports.

| **Variable** | **n=164** |
| --- | --- |
| **Donor characteristics** |  |
| Age, years | 56.0 (49.2-62.0) |
| Male, n (%) | 90 (54.9) |
| White, n (%) | 93 (56.7) |
| Arterial hypertension, n (%) | 128 (78.0) |
| Cause of brain death, n (%) |  |
| Stroke | 124 (75.6) |
| Trauma | 26 (15.9) |
| Anoxia | 12 (7.3) |
| Other | 2 (1.2) |
| Terminal serum creatinine, mg/dL | 1.58 (1.00-2.48) |
| KDPI, % | 89.5 (81.0-97.0) |
| KDRI | 1.59 (1.39-1.94) |
| ECD, n (%) | 116 (70.7) |
| **Recipient characteristics** |  |
| Age, years | 50.2 (39.9-59.1) |
| Male, n (%) | 103 (62.8) |
| White, n (%) | 80 (48.8) |
| CKD etiology, n (%) |  |
| Arterial hypertension | 87 (53.0) |
| Glomerulonephritis | 24 (14.6) |
| Diabetes mellitus | 23 (14.0) |
| ADPKD | 16 (9.8) |
| Urological | 9 (5.5) |
| Lithiasis | 4 (2.4) |
| Undetermined | 1 (0.6) |
| RRT duration, months | 36.9 (19.1-71.2) |
| Hemodialysis as RRT, n (%) | 155 (94.5) |
| Retransplantation, n (%) | 7 (4.3) |
| PRA, n (%) |  |
| Class I >0% | 34 (20.7) |
| Class II >0% | 19 (11.6) |
| Positive CMV IgG, n (%) | 153 (93.3) |
| **Transplant characteristics** |  |
| Zero HLA-A mismatch, n (%) | 19 (11.6) |
| Zero HLA-B mismatch, n (%) | 32 (19.5) |
| Zero HLA-DR mismatch, n (%) | 148 (90.2) |
| CIT, hours | 23.0 (19.0-27.8) |
| Thymoglobulin induction, n (%) | 163 (99.4) |
| **Immunosuppressive regimen, n (%)** |  |
| TAC/MPA | 114 (69.5) |
| TAC/AZA | 31 (18.9) |
| TAC/mTORi | 18 (11.0) |
| Other | 1 (0.6) |

**Abbreviations:** ADPKD, autosomal dominant polycystic kidney disease; AZA, azathioprine; CIT, cold ischemia time; CKD, chronic kidney disease; CMV, cytomegalovirus; ECD, expanded criteria donor; HLA, human leukocyte antigen; KDPI, Kidney Donor Profile Index; KDRI, Kidney Donor Risk Index; MPA, mycophenolic acid; mTORi, mammalian target of rapamycin inhibitor; PRA, panel reactive antibody; RRT, renal replacement therapy; TAC, tacrolimus.

**Supplementary Table 2.** Pre-implantation biopsy findings according to the Banff classification.

| **Banff score** | **Total (n=164)** |
| --- | --- |
| **Glomerular basement membrane thickening (cg), n (%)** |  |
| 0 | 164 (100.0) |
| 1 | 0 (0.0) |
| 2 | 0 (0.0) |
| 3 | 0 (0.0) |
| **Mesangial matrix expansion (mm), n (%)** |  |
| 0 | 161 (98.2) |
| 1 | 2 (1.2) |
| 2 | 1 (0.6) |
| 3 | 0 (0.0) |
| **Tubular atrophy (ct), n (%)** |  |
| 0 | 116 (70.7) |
| 1 | 45 (27.4) |
| 2 | 3 (1.8) |
| 3 | 0 (0.0) |
| **Interstitial fibrosis (ci), n (%)** |  |
| 0 | 114 (69.5) |
| 1 | 48 (29.3) |
| 2 | 2 (1.2) |
| 3 | 0 (0.0) |
| **Vascular fibrous intimal thickening (cv), n (%)** |  |
| 0 | 60 (36.6) |
| 1 | 73 (44.5) |
| 2 | 30 (18.3) |
| 3 | 1 (0.6) |
| **Arteriolar hyalinosis (ah), n (%)** |  |
| 0 | 67 (40.9) |
| 1 | 76 (46.3) |
| 2 | 21 (12.8) |
| 3 | 0 (0.0) |

**Abbreviations:** n, number.

**Supplementary Table 3.** Donor characteristics according to 5-year eGFR outcome.

|  | **eGFR <30 mL/min/1.73 m² at 5 years** | | | |
| --- | --- | --- | --- | --- |
| **Variable** | **Total (n=164)** | **No (n=81)** | **Yes (n=83)** | **p** |
| Age, years | 56.0 (49.2-62.0) | 55.0 (48.5-61.0) | 58.0 (50.0-62.0) | 0.17 |
| Male, n (%) | 90 (54.9) | 47 (58.0) | 43 (51.8) | 0.42 |
| White, n (%) | 93 (56.7) | 43 (53.1) | 50 (60.2) | 0.35 |
| Arterial hypertension, n (%) | 128 (78.0) | 62 (76.5) | 66 (79.5) | 0.64 |
| Cause of brain death, n (%) |  |  |  | 0.73 |
| Stroke | 124 (75.6) | 61 (75.3) | 63 (75.9) |  |
| Trauma | 26 (15.9) | 13 (16.0) | 13 (15.7) |  |
| Anoxia | 12 (7.3) | 6 (7.4) | 6 (7.2) |  |
| Other | 2 (1.2) | 1 (1.2) | 1 (1.2) |  |
| KDPI, % | 89.5 (81.0-97.0) | 89.0 (78.5-96.0) | 92.0 (82.0-98.0) | 0.12 |
| KDRI | 1.59 (1.39-1.94) | 1.56 (1.33-1.88) | 1.64 (1.40-2.10) | 0.10 |
| Terminal serum creatinine, mg/dL | 1.58 (1.00-2.48) | 1.50 (0.95-2.66) | 1.60 (1.00-2.30) | 0.92 |
| ECD, n (%) | 116 (70.7) | 57 (70.4) | 59 (71.1) | 0.92 |

**Abbreviations:** ECD, expanded criteria donor; eGFR, estimated glomerular filtration rate; KDPI, Kidney Donor Profile Index; KDRI, Kidney Donor Risk Index.

**Supplementary Table 4. Recipient characteristics according to 5-year eGFR outcome.**

|  | **eGFR <30 mL/min/1.73 m² at 5 years** | | | |
| --- | --- | --- | --- | --- |
| **Variable** | **Total (n=164)** | **No (n=81)** | **Yes (n=83)** | **p** |
| Age, years | 50.2 (39.2-58.6) | 49.2 (39.9-56.2) | 51.0 (39.9-61.9) | 0.15 |
| Male, n (%) | 103 (62.8) | 49 (60.5) | 54 (65.1) | 0.54 |
| White, n (%) | 80 (48.8) | 42 (51.9) | 38 (45.8) | 0.54 |
| CKD etiology, n (%) |  |  |  |  |
| Arterial hypertension | 87 (53.0) | 45 (55.6) | 42 (50.6) |  |
| Glomerulonephritis | 24 (14.6) | 14 (17.3) | 10 (12.0) |  |
| Diabetes mellitus | 23 (14.0) | 9 (11.1) | 14 (16.9) |  |
| ADPKD | 16 (9.8) | 7 (8.6) | 9 (10.8) |  |
| Urological | 9 (5.5) | 3 (3.7) | 6 (7.2) |  |
| Lithiasis | 4 (2.4) | 3 (3.7) | 1 (1.2) |  |
| Undetermined | 1 (0.6) | 0 (0.0) | 1 (1.2) |  |
| RRT duration, months | 36.9 (19.1-71.2) | 36.0 (17.1-69.5) | 44.7 (21.4-77.7) | 0.13 |
| Hemodialysis as RRT, n (%) | 155 (94.6) | 74 (91.4) | 81 (97.6) | 0.17 |
| Retransplantation, n (%) | 7 (4.3) | 3 (3.7) | 4 (4.8) | 0.73 |
| PRA class I >0%, n (%) | 34 (20.7) | 13 (16.0) | 21 (25.3) | 0.14 |
| PRA class II >0%, n (%) | 19 (11.6) | 9 (11.1) | 10 (12.0) | 0.85 |
| Positive CMV IgG, n (%) | 153 (93.3) | 72 (88.9) | 81 (97.6) | 0.03 |

**Abbreviations:** ADPKD, autosomal dominant polycystic kidney disease; CKD, chronic kidney disease; CMV, cytomegalovirus; eGFR, estimated glomerular filtration rate; IgG, immunoglobulin G; PRA, panel reactive antibody; RRT, renal replacement therapy.

**Supplementary Table 5.** Transplant and immunosuppression characteristics according to 5-year eGFR outcome.

|  | **eGFR <30 mL/min/1.73 m² at 5 years** | | | |
| --- | --- | --- | --- | --- |
| **Variable** | **Total (n=164)** | **No (n=81)** | **Yes (n=83)** | **p** |
| Zero HLA-A mismatch, n (%) | 19 (11.6) | 11 (13.6) | 8 (9.6) | 0.40 |
| Zero HLA-B mismatch, n (%) | 32 (19.5) | 18 (22.2) | 14 (16.9) | 0.45 |
| Zero HLA-DR mismatch, n (%) | 148 (90.2) | 74 (91.3) | 74 (89.2) | 0.45 |
| CIT, hours | 23.0 (19.0-27.8) | 23.0 (18.0-27.0) | 23.0 (20.0-29.0) | 0.53 |
| Thymoglobulin induction, n (%) | 163 (99.4) | 80 (98.8) | 83 (100.0) | 0.31 |
| **Immunosuppressive regimen, n (%)** |  |  |  | 0.07 |
| TAC/MPA | 114 (69.5) | 61 (75.3) | 53 (63.9) | 0.11 |
| TAC/AZA | 31 (18.9) | 16 (19.8) | 15 (18.1) | 0.78 |
| TAC/mTORi | 18 (11.0) | 4 (4.9) | 14 (16.9) | 0.01 |
| Other | 1 (0.6) | 0 (0.0) | 1 (1.2) | 0.32 |

**Abbreviations:** AZA, azathioprine; CIT, cold ischemia time; eGFR, estimated glomerular filtration rate; HLA, human leukocyte antigen; MPA, mycophenolic acid; mTORi, mammalian target of rapamycin inhibitor; TAC, tacrolimus.

**Supplementary Table 6.** Pre-implantation biopsy findings according to 5-year eGFR outcome.

|  | **eGFR <30 mL/min/1.73 m² at 5 years** | | | |
| --- | --- | --- | --- | --- |
| **Variable** | **Total (n=164)** | **No (n=81)** | **Yes (n=83)** | **p** |
| **Glomerular basement membrane thickening (cg), n (%)** |  |  |  | NA |
| 0 | 164 (100.0) | 81 (100.0) | 83 (100.0) |  |
| **Mesangial matrix expansion (mm), n (%)** |  |  |  | 0.21 |
| 0 | 161 (98.2) | 78 (96.3) | 83 (100.0) |  |
| 1 | 2 (1.2) | 2 (2.5) | 0 (0.0) |  |
| 2 | 1 (0.6) | 1 (1.2) | 0 (0.0) |  |
| 3 | 0 (0.0) | 0 (0.0) | 0 (0.0) |  |
| **Tubular atrophy (ct), n (%)** |  |  |  | 0.17 |
| 0 | 116 (70.7) | 62 (76.5) | 54 (65.1) |  |
| 1 | 45 (27.4) | 17 (21.0) | 28 (33.7) |  |
| 2 | 3 (1.8) | 2 (2.5) | 1 (1.2) |  |
| 3 | 0 (0.0) | 0 (0.0) | 0 (0.0) |  |
| **Interstitial fibrosis (ci), n (%)** |  |  |  | 0.27 |
| 0 | 114 (69.5) | 61 (75.3) | 53 (63.9) |  |
| 1 | 48 (29.3) | 19 (23.5) | 29 (34.9) |  |
| 2 | 2 (1.2) | 1 (1.2) | 1 (1.2) |  |
| 3 | 0 (0.0) | 0 (0.0) | 0 (0.0) |  |
| **Vascular fibrous intimal thickening (cv), n (%)** |  |  |  | 0.65 |
| 0 | 60 (36.6) | 30 (37.0) | 30 (36.1) |  |
| 1 | 73 (44.5) | 38 (46.9) | 35 (42.2) |  |
| 2 | 30 (18.3) | 13 (16.0) | 17 (20.5) |  |
| 3 | 1 (0.6) | 0 (0.0) | 1 (1.2) |  |
| **Arteriolar hyalinosis (ah), n (%)** |  |  |  | 0.26 |
| 0 | 67 (40.9) | 36 (44.4) | 31 (37.3) |  |
| 1 | 76 (46.3) | 38 (46.9) | 38 (45.8) |  |
| 2 | 21 (12.8) | 7 (8.6) | 14 (16.9) |  |
| 3 | 0 (0.0) | 0 (0.0) | 0 (0.0) |  |

**Abbreviations:** eGFR, estimated glomerular filtration rate; NA, not applicable.

**Supplementary Table 7.** Donor characteristics according to 5-year death-censored graft loss.

|  | **5-year death-censored graft loss** | | | |
| --- | --- | --- | --- | --- |
| **Variable** | **Total (n=164)** | **No (n=137)** | **Yes (n=27)** | **p** |
| Age, years | 56.0 (49.2-62.0) | 55.0 (49.0-61.0) | 60.0 (52.0-62.0) | 0.097 |
| Male, n (%) | 90 (54.9) | 74 (54.0) | 16 (59.3) | 0.62 |
| White, n (%) | 93 (56.7) | 78 (56.9) | 15 (55.6) | 0.89 |
| Arterial hypertension, n (%) | 128 (78.0) | 103 (75.2) | 25 (92.6) | 0.05 |
| Cause of brain death, n (%) |  |  |  | 0.19 |
| Stroke | 124 (75.6) | 108 (78.8) | 16 (59.3) |  |
| Trauma | 26 (15.9) | 19 (13.9) | 7 (25.9) |  |
| Anoxia | 12 (7.3) | 8 (5.8) | 4 (14.8) |  |
| Other | 2 (1.2) | 2 (1.5) | 0 (0.0) |  |
| KDPI, % | 89.5 (81.0-97.0) | 89.0 (81.0-97.0) | 95.0 (83.0-98.0) | 0.15 |
| KDRI | 1.59 (1.39-1.94) | 1.56 (1.38-1.92) | 1.79 (1.45-2.10) | 0.13 |
| Terminal serum creatinine, mg/dL | 1.58 (1.00-2.48) | 1.58 (1.00-2.55) | 1.70 (1.30-2.23) | 0.81 |
| ECD, n (%) | 116 (70.7) | 94 (68.6) | 22 (81.5) | 0.18 |

**Abbreviations:** ECD, expanded criteria donor; KDPI, Kidney Donor Profile Index; KDRI, Kidney Donor Risk Index.

**Supplementary Table 8.** Recipient characteristics according to 5-year death-censored graft loss.

|  | **5-year death-censored graft loss** | | | |
| --- | --- | --- | --- | --- |
| **Variable** | **Total (n 164)** | **No (n=137)** | **Yes (n=27)** | **p** |
| Age, years | 50.2 (39.9-59.1) | 50.9 (42.4-60.1) | 48.2 (39.3-55.0) | 0.11 |
| Male, n (%) | 103 (62.8) | 86 (62.8) | 17 (63.0) | 0.98 |
| White, n (%) | 80 (48.8) | 70 (51.1) | 10 (37.0) | 0.38 |
| CKD etiology, n (%) |  |  |  |  |
| Arterial hypertension | 87 (53.0) | 74 (54.0) | 13 (48.1) |  |
| Glomerulonephritis | 24 (14.6) | 20 (14.6) | 4 (14.8) |  |
| Diabetes mellitus | 23 (14.0) | 22 (16.1) | 1 (3.7) |  |
| ADPKD | 16 (9.8) | 12 (8.8) | 4 (14.8) |  |
| Urological | 9 (5.5) | 5 (3.6) | 4 (14.8) |  |
| Lithiasis | 4 (2.4) | 3 (2.2) | 1 (3.7) |  |
| Undetermined | 1 (0.6) | 1 (0.7) | 0 (0.0) |  |
| RRT duration, months | 36.9 (19.1-71.2) | 36.0 (18.5-69.9) | 42.8 (23.3-93.1) | 0.41 |
| Hemodialysis as RRT, n (%) | 155 (94.5) | 129 (94.2) | 26 (96.3) | 0.49 |
| Retransplantation, n (%) | 7 (4.3) | 7 (5.1) | 0 (0.0) | 0.23 |
| PRA class I >0%, n (%) | 34 (20.7) | 28 (20.4) | 6 (22.2) | 0.83 |
| PRA class II >0%, n (%) | 19 (11.6) | 17 (12.4) | 2 (7.4) | 0.46 |
| Positive CMV IgG, n (%) | 153 (93.3) | 128 (93.4) | 25 (92.6) | 0.87 |

**Abbreviations:** ADPKD, autosomal dominant polycystic kidney disease; CKD, chronic kidney disease; CMV, cytomegalovirus; IgG, immunoglobulin G; PRA, panel reactive antibody; RRT, renal replacement therapy.

**Supplementary Table 9.** Transplant and immunosuppression characteristics according to 5-year death-censored graft loss.

|  | **5-year death-censored graft loss** | | | |
| --- | --- | --- | --- | --- |
| **Variable** | **Total (n=164)** | **No (n=137)** | **Yes (n=27)** | **p** |
| Zero HLA-A mismatch, n (%) | 19 (11.6) | 17 (12.4) | 2 (7.4) | 0.56 |
| Zero HLA-B mismatch, n (%) | 32 (19.5) | 29 (21.2) | 3 (11.1) | 0.36 |
| Zero HLA-DR mismatch, n (%) | 148 (90.2) | 124 (90.5) | 24 (88.9) | 0.84 |
| CIT, hours | 23.0 (19.0-27.8) | 23.0 (18.5-27.0) | 22.0 (20.0-31.0) | 0.79 |
| Thymoglobulin induction, n (%) | 163 (99.4) | 136 (99.3) | 27 (100.0) | 0.65 |
| Immunosuppressive regimen, n (%) |  |  |  | 0.06 |
| TAC/MPA | 114 (69.5) | 94 (68.6) | 20 (74.1) |  |
| TAC/AZA | 31 (18.9) | 29 (21.2) | 2 (7.4) |  |
| TAC/mTORi | 18 (11.0) | 13 (9.5) | 5 (18.5) |  |
| Other | 1 (0.6) | 1 (0.7) | 0 (0.0) |  |

**Abbreviations:** AZA, azathioprine; CIT, cold ischemia time; HLA, human leukocyte antigen; MPA, mycophenolic acid; mTORi, mammalian target of rapamycin inhibitor; TAC, tacrolimus.

**Supplementary Table 10.** Pre-implantation biopsy findings according to 5-year death-censored graft loss.

|  | **5-year death-censored graft loss** | | | | | | | |
| --- | --- | --- | --- | --- | --- | --- | --- | --- |
| **Variable** | **Total (n = 164)** | | **No (n = 137)** | | **Yes (n = 27)** | | **p** | |
| **Glomerular basement membrane thickening (cg), n (%)** |  |  | |  | | NA | |  |
| 0 | 164 (100.0) | | 137 (100.0) | | 27 (100.0) | |  | |
| **Mesangial matrix expansion (mm), n (%)** |  | |  | |  | | 0.74 | |
| 0 | 161 (98.2) | | 134 (97.8) | | 27 (100.0) | |  | |
| 1 | 2 (1.2) | | 2 (1.5) | | 0 (0.0) | |  | |
| 2 | 1 (0.6) | | 1 (0.7) | | 0 (0.0) | |  | |
| 3 | 0 (0.0) | | 0 (0.0) | | 0 (0.0) | |  | |
| **Tubular atrophy (ct), n (%)** |  | |  | |  | | 0.72 | |
| 0 | 116 (70.7) | | 97 (70.8) | | 19 (70.4) | |  | |
| 1 | 45 (27.4) | | 37 (27.0) | | 8 (29.6) | |  | |
| 2 | 3 (1.8) | | 3 (2.2) | | 0 (0.0) | |  | |
| 3 | 0 (0.0) | | 0 (0.0) | | 0 (0.0) | |  | |
| **Interstitial fibrosis (ci), n (%)** |  | |  | |  | | 0.82 | |
| 0 | 114 (69.5) | | 95 (69.3) | | 19 (70.4) | |  | |
| 1 | 48 (29.3) | | 40 (29.2) | | 8 (29.6) | |  | |
| 2 | 2 (1.2) | | 2 (1.5) | | 0 (0.0) | |  | |
| 3 | 0 (0.0) | | 0 (0.0) | | 0 (0.0) | |  | |
| **Vascular fibrous intimal thickening (cv), n (%)** |  | |  | |  | | 0.06 | |
| 0 | 60 (36.6) | | 48 (35.0) | | 12 (44.4) | |  | |
| 1 | 73 (44.5) | | 65 (47.4) | | 8 (29.6) | |  | |
| 2 | 30 (18.3) | | 24 (17.5) | | 6 (22.2) | |  | |
| 3 | 1 (0.6) | | 0 (0.0) | | 1 (3.7) | |  | |
| **Arteriolar hyalinosis (ah), n (%)** |  | |  | |  | | 0.33 | |
| 0 | 67 (40.9) | | 53 (38.7) | | 14 (51.9) | |  | |
| 1 | 76 (46.3) | | 67 (48.9) | | 9 (33.3) | |  | |
| 2 | 21 (12.8) | | 17 (12.4) | | 4 (14.8) | |  | |
| 3 | 0 (0.0) | | 0 (0.0) | | 0 (0.0) | |  | |

**Abbreviations:** NA, not applicable.

**Supplementary Table 11.** Multivariable analysis of factors associated with 5-year death-censored graft loss among recipients of kidneys from donors with diabetes mellitus.

| **Variable** | **HR** | **95% CI** | **p** |
| --- | --- | --- | --- |
| Donor hypertension | 4.13 | 0.93-18.34 | 0.06 |

**Abbreviations:** CI, confidence interval; HR, hazard ratio.

**Note:** Variables removed by backward stepwise selection: vascular fibrous intimal thickening (cv) at step 2 and donor age at step 3.
